# CModel: An Informer-Based Model for Robust Molecular Communication Signal Detection

**DOI:** 10.3390/s25175453

**Published:** 2025-09-03

**Authors:** Wenxin Zhao, Pengfei Lu, Hui Sun, Pengfei Zhang, Xiaofang Wang

**Affiliations:** College of Information Science and Technology, Shihezi University, Shihezi 832003, China; 20232108001@stu.shzu.edu.cn (W.Z.); 20232108002@stu.shzu.edu.cn (P.Z.)

**Keywords:** Informer-based, molecular communication, probSparse Attention, Cross Attention, bit error rate, multi-noise

## Abstract

Molecular communication signal detection faces numerous challenges, including complex environments, multi-source noise, and signal drift. Traditional methods rely on precise mathematical models, which are constrained by drift speed and signal-to-noise ratio. To address these issues, this paper proposes an innovative detection model based on the Informer architecture, named ComModel (CModel). This framework integrates probSparse Attention, Cross Attention, and convolutional layers to enhance detection accuracy and adaptability to various environmental conditions. Experimental results demonstrate that CModel consistently outperforms traditional deep neural networks and Transformer-based models, especially in complex scenarios with varying drift speeds and noise levels. As the drift speed increases, CModel maintains superior stability and exhibits lower bit error rates, particularly at medium and high drift speeds. Moreover, CModel shows excellent performance in environments with significant noise. Overall, CModel demonstrates robust and reliable signal detection capabilities in multi-noise environments.

## 1. Introduction

As an emerging communication technology, molecular communication (MC) has attracted considerable attention in recent years. Its communication mechanism draws inspiration from processes such as signal transduction and molecular recognition between cells in nature, enabling information exchange within living organisms and among microscopic systems in vitro.

With the advancement of nanotechnology and microscale sensors, MC, serving as a bridge between nano-devices and biological systems, exhibits significant application potential in various fields including healthcare, intelligent diagnostics, environmental monitoring, and military security. For instance, in implanted sensor networks and targeted drug delivery systems, MC enables real-time monitoring and precise control of key physiological parameters [1]. Additionally, MC plays a vital role in the realization of highly integrated and cooperative networks of biosensors and actuators, and in enhancing the effectiveness of telemedicine and precision diagnostics [2].

Compared to traditional electromagnetic wave communication, MC in nanoscale communication offers three main advantages. First, low energy consumption is one of the fundamental requirements for nanoscale communication, and MC is more energy-efficient than traditional methods. Second, MC can reduce antenna sizes, a capability that has been validated in several natural instances [3]. Third, when employing harmless molecules, MC exhibits superior biocompatibility within the body compared to traditional methods [4]. In response to the varying transmission requirements of MC channels, several modulation schemes have been proposed and implemented. For example, On–Off Keying (OOK) modulation is a simple and effective method used to transmit information depending on the presence or absence of molecular signals [5]. Molecule Shift Keying (MoSK) modulation encodes information by changing the types of released molecules, thereby enhancing the system’s expressive capability [6]. Another modulation approach is Depleted MoSK (D-MoSK), which introduces a molecular consumption mechanism based on traditional MoSK to improve signal discernibility and anti-interference performance [7]. Each of these modulation schemes has its own characteristics, providing multiple options for the design and optimization of MC systems.

The transmission process of MC is influenced by various physical factors, such as diffusion, signal attenuation, and noise, which significantly affect communication performance [7]. An in-depth study of these physical characteristics will provide guidance for optimizing the design and performance of MC systems.

Bit error rate (BER) is an important metric for assessing the efficiency and reliability of communication systems, reflecting the proportion of erroneous bits in the data transmission process. Minimizing the BER is equivalent to enhancing transmission efficiency and signal fidelity. Therefore, studying the BER and its determining factors in MC is vital for optimizing system performance.

Traditional wireless communication systems are typically modeled using linear paradigms, which approximate certain nonlinear elements in signal transmission through the cascading of linear modules. However, this strategy based on linear assumptions has inherent flaws, rendering it incapable of accurately reproducing the diverse nonlinear effects in real-world communication environments, thereby limiting the comprehensive enhancement of system performance. Furthermore, MC involves numerous random chemical mechanisms, making it difficult for traditional linear mathematical tools to fully depict its dynamic essence and consequently reducing the reliability of signal interpretation.

In recent years, deep learning (DL), as a core component of artificial intelligence (AI), has garnered widespread attention and practical application in the field of wireless communications due to its outstanding performance in modeling and optimizing complex systems [8,9]. Despite these advancements, existing deep learning detection methods in MC scenarios still exhibit significant shortcomings. Researchers have attempted to leverage deep learning to extract patterns from data [10,11], such as employing neural networks (NN) for signal detection [10] and utilizing vanilla recurrent neural networks (vanilla RNN) for detection and decoding [11]. However, these models are relatively simple in design, making it challenging to effectively capture the complex dynamic characteristics of MC, particularly the impact of inter-symbol interference (ISI), thereby resulting in detection accuracy that falls short of ideal levels.

To address the aforementioned challenges, this paper proposes a novel Informer-based detector for performing signal detection in the MC system. The primary contributions of this paper are as follows:

1. We propose a novel receiver model, CModel, based on the Informer architecture, which effectively mitigates ISI in the communication channel, thereby significantly reducing BER.
2. We demonstrate that CModel achieves lower BER than Transformer- and DNN-based detectors across varying drift velocities and noise levels, confirming superior robustness.
3. We confirm that CModel consistently preserves low BER under diverse signal-to-noise ratio (SNR) conditions, ensuring stable and reliable detection in noisy environments.

The structure of this paper is as follows: Section 2 presents an overview of related works, and Section 3 primarily describes the MC system model and the structure of CModel. Section 4 presents and analyzes the experimental results of CModel. Section 5 discusses the performance advantages and limitations of CModel. Section 6 summarizes the main contributions of this research.

## 2. Related Work

In this context, compared to traditional methods, deep learning can leverage its powerful nonlinear modeling capabilities to capture both linear and nonlinear components within communication systems, providing accurate and comprehensive system descriptions. This paper applies deep learning to the design of MC system receivers, taking full advantage of its strengths in nonlinear feature recognition and model construction to enhance signal demodulation performance and the overall communication quality of the system. Deep learning algorithms such as Informer, Transformer, and DNN can effectively extract complex correlations from data and autonomously learn patterns present in the communication process.

To enhance the detection performance of MC system receivers, researchers have begun to utilize deep learning techniques for signal detection. Yue et al. [12] derived a mathematical model for the channel impulse response based on microcirculation networks (MCNs) and blood flow characteristics, further constructing an end-to-end communication model based on MCNs and analyzing the probability of error. Farsad et al. [13] proposed a Sliding Bidirectional Recurrent Neural Network (SBRNN) detection technique that can estimate the received signal flow in real time through training without relying on an underlying channel model. Experimental results indicate that SBRNN performs well under various channel conditions, achieving lower BER than detectors with imperfect channel state information and other neural network detectors, demonstrating its effective adaptation to rapidly changing channel environments.

Gomez et al. [14] proposed an interpretable deep learning symbol detection method based on real test platform data. Bartunik et al. [15] designed a signal demodulation method using convolutional neural networks, suitable for scenarios with varying channel parameters. This method requires minimal channel knowledge and achieved a classification accuracy of 1% at high symbol rates, significantly outperforming linear discriminant analysis, thereby demonstrating the potential of deep learning applications in MC. Additionally, an improved time series convolutional network has been introduced for signal detection in specific mobile communication systems [16]. Lee et al. [17] employed artificial neural networks to predict MC channel parameters and trained their model using nonlinear least squares curve fitting based on simulation data.

Baydas et al. [18] assessed the BER performance of MC systems using convolutional neural networks and recurrent neural networks. Subsequently, Transformer was also applied in the detection model, and the results indicated that its performance significantly surpassed that of DNN detectors [19,20]. Khalopour et al. [21] analyzed the performance of fusion centers and gateways under ideal (noise-free) and non-ideal (noisy) channel conditions concerning perception, communication, and localization issues in MC systems.

The related work is summarized in Table 1. These studies underscore the promise of deep learning for MC detection but also reveal persistent limitations in capturing nonlinear stochastic dynamics and severe ISI under varying drift and multi-source noise, motivating our Informer-based receiver, CModel, detailed in Section 3.

## 3. Methods

### 3.1. MC System Model

**Figure 1:**
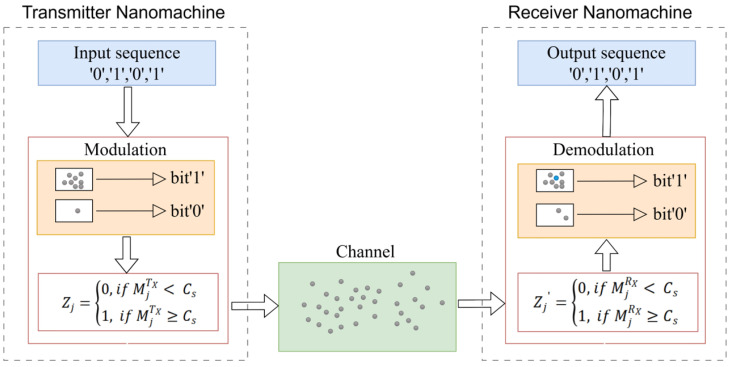
The workflow of the transmitter and receiver nanomachines in the MC system is depicted. On the left is the TN, which converts the input sequence ‘0’, ‘1’, ‘0’, ‘1’ into corresponding molecular signals during the modulation process. In the modulation module, the number of molecules transmitted depends on the bit value: if the bit is ‘0’, the number of molecules sent is either 0 or below the threshold $C_{s}$, if the bit is ‘1’, the number of molecules sent is $C_{s}$. On the right is the RN, which receives the transmitted molecular signals and generates the corresponding output sequence. The demodulation module determines the output bit value based on the number of molecules received: if the number of received molecules is below $C_{s}$, the output bit is ‘0’; otherwise, the output bit is ‘1’.

The fundamental components of MC system include the transmitter nanomachine (TN), the receiver nanomachine (RN), and the channel. Figure 1 illustrates a three-dimensional diffusion system model of a molecular single-input single-output (SISO) communication system, in which both the transmitter and receiver are static, demonstrating how the signal propagates through the molecular diffusion channel.

#### 3.1.1. Transmitter Model

The transmitter is modeled as TN in three-dimensional space, located at a specific distance from the receiver, and releases information molecules (IM) [22,23]. These molecules freely diffuse via Brownian motion and propagate independently and stochastically to RN [24], with each molecule’s diffusion behavior being independent and unaffected by other molecules. 

The design of the transmitter should take into account factors such as the type of molecules, release rate, and release method. Assuming that the transmitter releases $R\left(t\right)$ molecules at time t, the release rate $v\left(t\right)$ is expressed as

(1) $$v\left(t\right)=\frac{dR\left(t\right)}{dt}$$

Assuming that at time t=0, the transmitter releases $M^{T_{X}}$ molecules, the number of particles received by the receiver, ${R_{1}}^{R_{X}}$, can be regarded as a binomial random variable, which is specifically expressed as

(2) $${R_{1}}^{R_{X}}\sim Β\left(M^{T_{X}},Q_{1}\right)$$

The parameter $Q_{1}$ represents the expected probability that the receiver node absorbs molecules during the initial symbol period. The binomial distribution is typically denoted as Β(n,p). In the case of overlapping release events, the number of received molecules is influenced not only by the current release but also by the accumulation from previous releases. Therefore, the number of received molecules can be expressed as

(3) $${R_{i}}^{R_{X}}\sim \sum_{j=1}^{i}\left(B(M_{j}^{T_{X}},Q_{i-j+1})\right),$$

where $M_{j}^{T_{X}}$ denotes the number of molecules released during the j-th symbol duration, ${R_{i}}^{R_{X}}$ represents the random number of molecules received in the i-th time slot, and $Q_{i-j+1}$ indicates the absorption probability or molecular detection probability for the corresponding delay step. Due to the complexity of analyzing binomial random variables, the Gaussian approximation method is generally adopted for related calculations, which is expressed as [25]

(4) $${R_{i}}^{R_{X}}\sim N\left(\sum_{j=1}^{i}M_{j}^{T_{X}}Q_{i-j+1},\sum_{j=1}^{i}M_{j}^{T_{X}}Q_{i-j+1}(1-Q_{i-j+1})\right).$$

In the formula, the number of received molecules ${R_{i}}^{R_{X}}$ is influenced not only by the current time instant but also by interference from continuous data transmissions at previous instants. Concentration Shift Keying (CSK) is a widely used modulation technique in MC systems. This modulation method adjusts the concentration keying such that the number of IM released per unit volume serves to carry and transmit data, thereby enabling the encoding and transmission of information [23,26,27,28,29,30,31]. This encoding approach, based on variations in molecular concentration, provides an effective means of information representation in MC systems.

Binary Concentration Shift Keying (BCSK) is adopted as the modulation scheme to analyze decoding performance [32]. In BCSK, the transmission of symbol “1” corresponds to a molecular concentration of $C_{s}$, while symbol “0” corresponds to zero concentration. Molecules reach the receiver through free diffusion. For the Gaussian model, the BCSK decision threshold is defined as follows:

(5) $$Z_{j}=\left\{\begin{array}{c} 0,\;\;\;\;if\;M_{j}^{T_{X}}<\;C_{s},\\ 1,\;\;\;\;if\;M_{j}^{T_{X}}\geq C_{s}, \end{array}\right.$$

where $M_{j}^{T_{X}}$ and $Z_{j}$ denote the number of molecules released during the j-th symbol duration and the corresponding modulation symbol, respectively, and $C_{s}$ is the decision threshold.

#### 3.1.2. Channel Model

Molecular transport typically occurs in free-diffusion media, such as air [33]. In the medium, molecules rely on thermal kinetic energy to constantly collide with molecules of the medium, thereby achieving random diffusion. More generally, the transmission medium refers to the propagation environment between the transmitter and receiver, which is usually a liquid or gas in a continuous spatial state characterized by random molecular motion. The physical properties of the medium, such as the diffusion coefficient D and temperature, directly influence the diffusion rate and direction of the molecules.

The variations in molecular concentration over time and space are described by the classical Fick’s law of diffusion, which is expressed as a partial differential equation:

(6) $$\frac{\partial C\left(x,t\right)}{\partial t}=D\nabla^{2}C\left(x,t\right),$$

where $C\left(x,t\right)$ represents the molecular concentration at position x and time t; D is the diffusion coefficient, which reflects the constraints imposed by the medium on molecular diffusion. The selection of signaling molecules should take into account their stability, reaction rate, and biocompatibility. In certain applications, such as fluorescence detection, the temporal variation in signaling molecule concentration is also influenced by chemical reactions, which can typically be described by first-order reaction kinetics:

(7) $$\frac{dc\left[S\right]}{dt}=-kc\left[S\right],$$

where $c\left[S\right]$ denotes the concentration of signaling molecules, and *k* is the reaction rate constant representing the rate of concentration decay. To accurately simulate the spatial movement of individual molecules, it is necessary to track their three-dimensional coordinates $\left(x_{i},y_{i},z_{i}\right)$, which are updated over time. The displacement of each particle can be represented as the superposition of its current position and its position at the previous time step [19]:

(8) $$\left(x_{i},y_{i},z_{i}\right)=(x_{t-∆t},y_{t-∆t},z_{t-∆t})\;+\;(∆x,∆y,∆z),$$

where ∆t denotes the discrete time interval, $\left(x_{t-∆t},y_{t-∆t},z_{t-∆t}\right)$ represents the position of the particle at the previous time instant, and (∆x,∆y,∆z) denotes the incremental displacement during the interval ∆t. Owing to the characteristics and flow of the medium, the incremental displacement can be modeled as a normal distribution with a specific mean and variance. Assuming a velocity v along the x-axis, the expected displacement of the particle in this direction is given by [1]:

(9) ∆x~N(v∆t,2D∆t),

where the mean value v∆t characterizes the average advection effect of velocity on particle displacement, while the variance 2D∆t reflects the random fluctuations induced by diffusion. Conversely, in the y and z directions, which are perpendicular to the velocity, there is no steady flow; the net expected displacement of the particles is zero, and the increments follow a normal distribution with zero mean:

(10) ∆y,∆z~Ν(0,2D∆t).

The variance reflects the stochasticity due to diffusion, indicating that D determines the motional characteristics of particles in the non-flow directions. In summary, by integrating diffusion equations, biochemical reaction kinetics, and stochastic motion models to describe the molecular transport process in a flowing medium, a theoretical foundation is established for subsequent studies.

#### 3.1.3. Receiver Model

In MC systems, the receiver is the core component responsible for detecting and demodulating the molecular signals emitted by the transmitter [34]. Within each symbol interval, the number of molecules detected by the receiver, $N_{j}$ can be approximated as following a normal distribution:

(11) $$N_{j}\sim N(\mu_{j},\sigma_{j}^{2})$$

where the mean value $\mu_{j}$ is calculated as follows:

(12) $$\mu_{j}=\int_{(j-1)T_{s}}^{jT_{s}}a\left(U(r,t)\right)dt.$$

In the formula, $T_{s}$ denotes the symbol interval length, and a(U(r,t)) represents receiver’s response to the molecular concentration signal at position r and time t. U(r,t) is the cumulative concentration of the signal at the receiver’s position *r*. If the integral of the cumulative concentration over the symbol interval exceeds the threshold $C_{s}$, the received signal is determined as symbol “1”; otherwise, it is determined as “0”. The value of t lies within the current symbol interval.

This channel is typically assumed to be a free diffusion channel in air. Ambient molecules, due to thermal motion, constantly collide with signal molecules, resulting in the random diffusion of the signal molecules. For each molecule, its distance to the center of the receiver is defined as

(13) $$r=\sqrt{{\left(x-x_{r}\right)}^{2}+{\left(y-y_{r}\right)}^{2}+{\left(z-z_{r}\right)}^{2}},$$

where $\left(x_{r}{,y}_{r},z_{r}\right)$ denotes the center position of the receiver. When the distance from a particle to the receiver is less than the receiver radius R, the particle is counted as a “hit” and the hit count is incremented:

(14) $$N_{hit}=\sum_{r<R}1,$$

this formula is used to count whether the particle has entered the receiver region. During the simulation, the trajectory and hitting status of each particle are recorded, resulting in stochastic signal samples in the time domain. Based on the diffusion equation and the absorbing receiver model, the probability density function for a particle arriving at the receiver can be theoretically calculated and is given by [1]:

(15) $$P_{hit}(t_{j})=\frac{R}{d}erfc\left(\frac{d-R-v_{j}t_{j}}{\sqrt{4Dt_{j}}}\right),$$

where erfc(·) is the complementary error function, $v_{j}$ represents the drift velocity during the *j*-th symbol interval, d is the distance between the transmitter and the receiver.

Within this theoretical framework, the expected number of particle hits can be expressed as

(16) $$N_{theoretical}(t_{j})=P_{hit}(t_{j})N_{hit}.$$

### 3.2. An Informer-Based Receiver Model—CModel

In this section, we introduce CModel, a model specifically designed for detecting emission signals, aimed at improving the accuracy and reliability of signal detection.

#### 3.2.1. Model Structure

**Figure 2:**
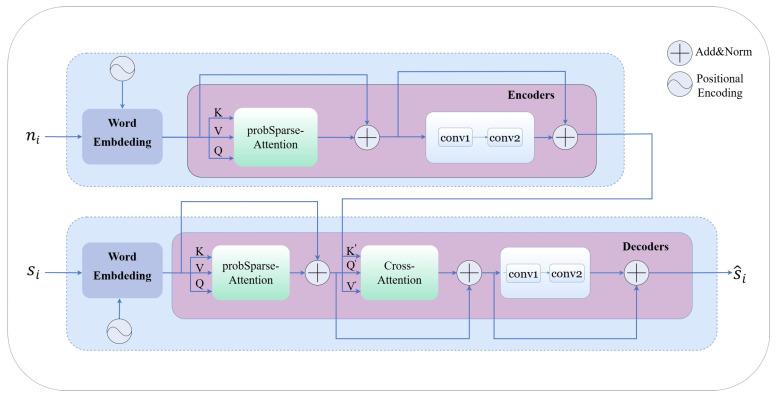
CModel network structure diagram. The encoder includes word embedding, probSparse Attention and convolutional layers. The decoder has a similar structure, including probSparse Attention, Cross Attention, and convolutional layers.

CModel is based on Informer and uses probSparse Attention, Cross Attention, and convolution layers to improve the performance of the model in sequence modeling. The overall structure of CModel is shown in Figure 2.

A. Word Embedding

The input consists of a sequence, and the source embedding layer maps the input sequence to a designated feature space using an embedding matrix. This mapping facilitates the transformation of sparse discrete data into dense vector representations while preserving semantic information. To incorporate positional information for each element in the input sequence, the model employs positional encoding, which is calculated as follows [35]:

(17) $$PE(pos,2i)\;=\;sin\left(\frac{pos}{10000^{\frac{2i}{d}}}\right),$$

(18) $$PE(pos,2i+1)=cos\left(\frac{pos}{10000^{\frac{2i}{d}}}\right),$$

where pos refers to the position index, i denotes the dimension index, and d refers to the embedding dimension. The final embedded representation is given by

(19) $$X=Embedding(n_{i})+PE(pos),$$

where X is the embedded representation, pos is the position index, and PE(pos) denotes the corresponding positional encoding vector.

B. Encoder

The encoder consists of three identical encoder layers, each containing a probSparse Attention and convolutional operations. The encoder initially receives X and then computes query Q, key K, and value V, with the calculation given by [35]:

(20) $$Q=XW^{Q},\;K=XW^{k},\;V=XW^{V},$$

where $W^{Q},W^{k},W^{V}$ represent the weights for the linear transformation.

Subsequently, Q, K, and V are input into the probSparse Attention layer, which optimizes the attention computation process through a probabilistic sparsification strategy. Specifically, this mechanism first calculates the dot product similarity between the queries and keys, then sparsifies the attention scores based on a probabilistic threshold, retaining only the most relevant key–value pairs for subsequent calculations. Next, the filtered scores undergo softmax normalization, and finally, the normalized attention weights are weighted and summed with the value vectors to produce the final attention output. The core computational process can be represented by Equation (21) [35]:

(21) $$Z=ProbAttn\left(Q,K,V\right)=softmax\left(\frac{QK^{T}}{\sqrt{d_{k}}}\right)V,$$

where $d_{k}$ indicates the dimensions of Q and K. The sparse matrix Q includes only the Top-u queries under the sparsity measurement, where u is typically derived from the product of the constant sampling factor c and the natural logarithm of $L_{Q}$, denoting the length of Q. Thus, probSparse Attention requires only $O(\ln L_{Q})$ dot products for each query–key lookup, leading to a memory usage of $O(L_{K}\ln L_{Q})$, where $L_{K}$ represents the length of K. In the context of the multi-head attention mechanism, this method generates different sparse query–key pairs for each head, thereby avoiding significant information loss. This strategy selects only the top-u most informative queries for attention computation, thereby significantly reducing redundant calculations. This reduces the complexity while still effectively preserving global dependencies without significant loss of crucial information. For MC signals that contain a lot of redundancy and noise, the ProbSparse attention mechanism helps the model focus on the most important temporal and statistical features, thereby improving the efficiency and quality of feature extraction and supporting downstream detection tasks.

The output of probSparse Attention first undergoes layer normalization and a residual connection, and then is input into a fully connected feedforward neural network, represented mathematically as:

(22) $$M=LayerNorm\left(Z+X\right).$$

Subsequently, the vector is processed through two linear layers, followed by a combination of residual connection and bias operation. The output of this process can be represented as:

(23) $$P=LayerNorm\left(Conv2\left(Conv1(M)\right)+M\right).$$

C. Decoder

The decoder is composed of three DecoderLayer components, with each layer containing probSparse Attention and Cross Attention. The input to the decoder is a sequence of features, which is passed through the embedding layer and position encoding to obtain the input representation $X^{\prime }$. Following Equation (20), the input representation $X^{\prime }$ is projected into Q, K, and V vectors through learnable linear transformations, which are then processed by probSparse Attention to capture sequence dependencies, as illustrated below:

(24) $$X_{1}=LayerNorm\left(X^{\prime }+ProbAttn\left(X^{\prime }\right)\right).$$

In the Cross Attention layer, the state $X_{1}$ within the decoder serves as the query input, while the keys come from the output L of the encoder. This interaction is first performed through a linear transformation. This enables the decoder to selectively focus on the most relevant features from the encoding stage in each decoding step, yielding:

(25) $$Q^{\prime }=X_{1}W_{c}^{Q},\;K^{\prime }=PW_{c}^{K},\;V^{\prime }=PW_{c}^{V}.$$

The Cross Attention is then calculated as follows:

(26) $$CrossAttn\left(X_{1},P\right)=softmax\left(\frac{Q^{\prime }K^{\prime T}}{\sqrt{d_{k}}}+M_{C}\right)V^{\prime },$$

where *P* denotes the output from the encoder, and $M_{C}$ represents the mask for Cross Attention, $K^{\prime }$ and $V^{\prime }$ are obtained from *P* through linear transformations using learnable weight matrices $W_{c}^{K}$ and $W_{c}^{V}$, respectively. The output is then normalized with a residual connection, as illustrated below:

(27) $$X_{2}=LayerNorm\left(X_{1}+CrossAttn\left(X_{1},P\right)\right).$$

The intermediate representation $X_{2}$ first undergoes two consecutive convolutional operations (*Conv*1 followed by *Conv*2). The result is then combined with the original input $X_{2}$ via residual connection, followed by layer normalization. This process is formulated as:

(28) $$Output=LayerNorm\left(Conv2\left(Conv1(X_{2})\right)+X_{2}\right).$$

CModel uses probSparse Attention, Cross Attention, and convolution layers. This model can efficiently handle tasks related to processing sequences.

#### 3.2.2. Data Generation

In this study, a signal generation model based on Brownian motion is employed to simulate particle migration in MC and to calculate the number of particles arriving at the receiver [36]. By incorporating free diffusion and several key physical parameters, this model enables the simulation of particle dynamics under specific environmental conditions. The model utilizes Brownian motion to generate signals for the particles and computes the probability of their arrival at the receiver under predefined configurations. During the simulation, the trajectories of each released particle are recorded, and the cumulative effect captures the synergistic interaction between Brownian motion and drift velocity. By calculating the number of particles received, the total number of hit molecules over the entire time series is obtained. Furthermore, the number of received molecules is decoded to recover the transmitted signal sequence.

In this study, the training data for the CModel is generated by simulating the MC process, with the specific steps as follows:

First, a random signal sequence $\left[s_{1},s_{2},{...,s}_{L}\right]$ is generated, where each element $s_{i}$ represents the symbol state transmitted in the i-th time slot, typically expressed as a binary value (such as 0 or 1). The sequence length is determined according to the requirements of the signal design, and is set to 100 bits in this work.

Subsequently, molecular release behavior is simulated based on the signal state $s_{i}$ of each time slot. When $s_{i}=1$, a predetermined number M of molecules are released; when $s_{i}=0$, zero or very few molecules are released. The timing of molecular release is strictly aligned with each time slot to ensure the temporal accuracy of the simulation.

After release, molecules propagate through the diffusion channel, and their mobility is mainly influenced by Brownian motion and drift. The model simulates the trajectory of each molecule and, based on the diffusion model, calculates the spatial distribution of molecules. At the receiver side, according to its physical size and spatial position, the number of molecules entering the receiving area in each time slot is counted and denoted as $n_{i}$, which reflects the response of the receiver to the transmitted signal. $n_{i}$ serves as the observed value at the receiver end, carrying the main information from $s_{i}$, but also being subject to the randomness and noise of the channel. Therefore, $n_{i}$ is the core intermediate variable through which the information of $s_{i}$ is conveyed to the receiver and is then used for signal detection.

The entire simulation process is repeated to generate a large number of signal sequences and their corresponding received molecule count sequences, thereby constructing the training and test datasets. Specifically, 9000 sets of random signal sequences are generated as the training and validation set (divided in a ratio of 7:2), and an additional 5000 sets are generated as the test set. Each sample contains the received molecule count sequence $\left[n_{1},n_{2},{...,n}_{100}\right]$, which serves as input to the CModel for model training and performance evaluation.

Through iterative simulation, the number of molecules arriving at the receiver under the specified conditions is computed. The trajectories of particle movement are ultimately stored, and the number of hits corresponding to the release signals is recorded. The parameters used in the simulation are listed in Table 2.

#### 3.2.3. Model Training

CModel is implemented using the PyTorch (2.3.1) framework and consists of an encoder with 3 encoder layers and a decoder with 3 decoder layers, equipped with 8 attention heads and a hidden layer dimension of 128. To mitigate the vanishing gradient problem, we employ the ReLU activation function and subsequently optimize the model using the Adam optimizer. To prevent overfitting, we set the dropout parameter to 0.1. The model was trained for 500 epochs with a batch size of 60 on an NVIDIA A100 GPU. Table 3 lists the key parameters of CModel, covering the structure and training configuration of the model.

In the training phase, the cross entropy function is used as the loss function. By quantifying the deviation between the prediction probability and real label, the loss function guides the model to adjust parameters continuously, so as to improve the accuracy and discrimination ability of classification boundary. In addition, the accuracy of the CModel was systematically evaluated to verify the accuracy of the model.

BER is selected as the core evaluation index to evaluate the performance of each model under different conditions. BER, as a key performance metric in communication system, can quantify the probability of error code generated by the model in the transmission process and reflect the accuracy of the model for signal transmission. By comparing BER performances of this model, MAP algorithm [10], DNN [16] and Transformer [16] model under different SNR environments, the adaptability of each model in multi-noise environment is evaluated.

## 4. Results

### 4.1. Model Accuracy

**Figure 3:**
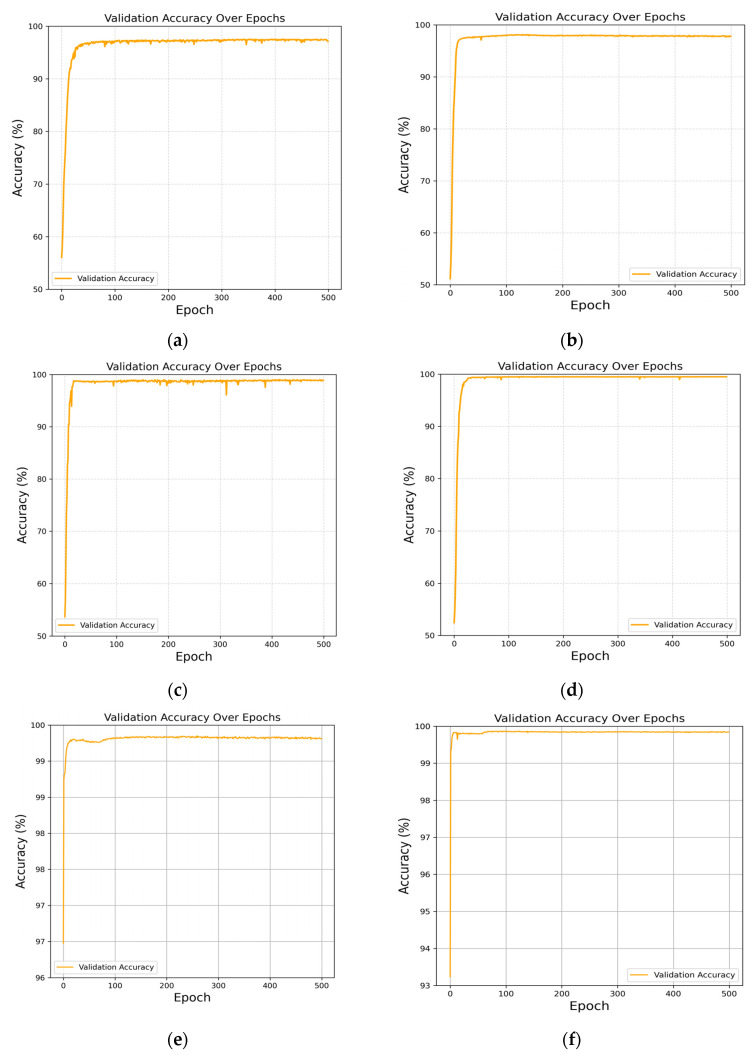
Accuracy performance of CModel at different velocities: (**a**) v=25 μm/s; (**b**) v=30 μm/s; (**c**) v=35 μm/s; (**d**) v=40 μm/s; (**e**) v=45 μm/s; (**f**) v=50 μm/s. The changes in the validation accuracy of CModel under different drift velocities are presented. The results demonstrate that, irrespective of the drift velocity, the model is able to converge rapidly and maintain high accuracy within a relatively short number of training epochs.

Figure 3a–f illustrate the changes in the validation accuracy of CModel during training under different drift velocities. The results indicate that, regardless of the drift velocity, the model can rapidly converge within a relatively short number of training epochs and maintain high accuracy, demonstrating strong robustness and adaptability to various drift velocity environments. Specifically, when the drift velocity is 25 μm/s, the model achieves an accuracy of over 93% after approximately 30 training epochs and remains stable in subsequent training. As the velocity increases to 30 μm/s, 35 μm/s, and 40 μm/s, the convergence speed of the model is significantly accelerated, with early-stage accuracy exceeding 95%. As training continues, the accuracy stabilizes and reaches nearly 100%. This indicates that higher drift velocities are more conducive to enabling the model to extract effective information from signals, thereby improving detection performance.

At higher drift velocities, such as 45 μm/s and 50 μm/s, the validation accuracy further increases to above 99%, and the training curves exhibit smoother and more stable behavior. This demonstrates that the model can fully leverage the dynamic characteristics of signals provided by high drift velocities, allowing it to recognize signals more accurately.

Overall, the trend reveals that increasing the drift velocity positively influences both the training efficiency and the accuracy of CModel, and the model exhibits strong generalization capability across different velocity ranges. The results highlight the suitability and efficiency of CModel for signal detection at various transmission velocities in MC systems, and demonstrate its potential for application in practical, complex, and multivariate channel environments.

### 4.2. BER Comparison of Different Models

We can obtain the decision signal corresponding to the transmitted signal $s_{i}$ from the received signal, denoted as ${\hat{s}}_{i}$. BER is calculated as follows [19]:

(29) $$BER=1-\frac{1}{L}\sum_{i=1}^{L}\delta ({\hat{s}}_{i},s_{i}),$$

where L denotes the length of the entire transmitted signal sequence, and the signal sequence is denoted by ${[s}_{1},s_{2},s_{3},...,s_{i}]$. The function $\delta ({\hat{s}}_{i},s_{i})$ is an indicator function: it takes 1 when the detected signal ${\hat{s}}_{i}$ is equal to the true signal $s_{i}$; otherwise it takes 0. This formula quantifies error detection performance by calculating error rate by comparing consistency between transmitted and detected signals.

Molecular signal generation was accomplished through multiple rounds of simulations modeling the Brownian motion of particles. By statistically analyzing the number of molecules successfully arriving at the receiver under different environmental conditions, the corresponding BER metrics were calculated.

**Figure 4:**
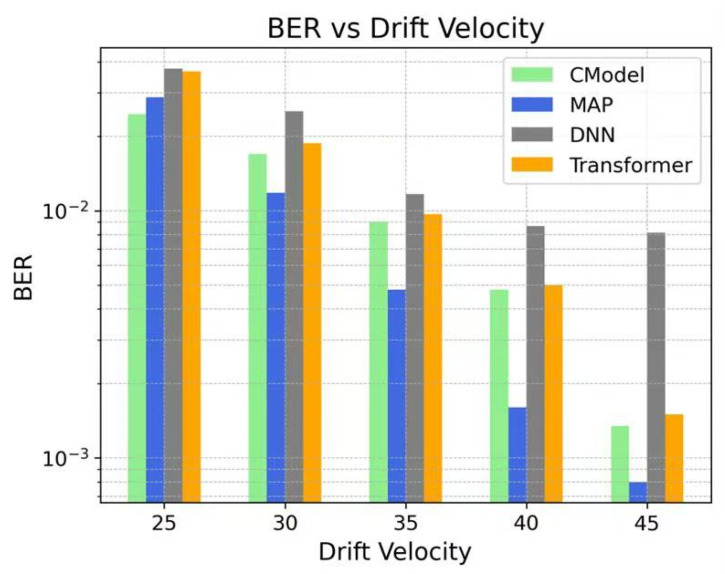
BER of CModel, MAP, DNN, and Transformer at various drift velocities. The results indicate that the BER of all models decreases with increasing drift velocity, among which the CModel demonstrates superior BER performance at most velocity points. Notably, in the medium-to-high drift velocity range, the BER of CModel is significantly lower than that of the conventional DNN and Transformer models.

In this study, the BER performance of various models was systematically evaluated at different drift velocities, and the performance of different detection methods in MC systems was further analyzed. Figure 4 illustrates the trend of BER for each model as the drift velocity varies. Experimental results indicate that the BER of all models decreases as the drift velocity increases, with the CModel exhibiting superior BER performance at most velocity conditions. Notably, in the medium-to-high drift velocity range, the CModel demonstrates more stable performance and a significantly lower BER compared to conventional DNN and Transformer models, highlighting its outstanding capability in suppressing channel interference.

### 4.3. BER Evaluation of Noise

In practical MC environments, possible unknown interferences include measurement errors (such as inaccurate counting of molecules at the receiver), external interferences (e.g., random molecular flows in biological bodies, temperature changes, or other chemical reactions), and channel uncertainties (due to Brownian motion and flow drift, which render molecular propagation inherently random). To further evaluate the robustness of the model against these unknown channel noise, this paper simulates external unknown interferences by adding additive white Gaussian noise. Specifically, additive white noise is introduced into the test signal sequence $\left[n_{1},n_{2},...,n_{L}\right]$. By superimposing Gaussian white noise on the source signal, signal data with different SNRs are generated. The new input signal sequence is denoted as $\left[n_{1}^{\prime },n_{2}^{\prime },...,n_{L}^{\prime }\right]$. Based on the power of the original signal, its average power Ρ is calculated as follows:

(30) $$Ρ=\frac{1}{L}\sum_{i=1}^{L}{n_{i}}^{2}$$

Based on a given SNR, the variance of the noise is defined as

(31) $$\sigma^{2}=\frac{Ρ}{10^{\left(\frac{SNR}{10}\right)}}$$

Gaussian noise is superimposed onto the signal, yielding a noise-corrupted signal denoted as

(32) $$\omega \sim N\left(0,\sigma^{2}\right)$$

(33) $${n_{i}}^{\prime }=n_{i}+\omega_{i}$$

**Figure 5:**
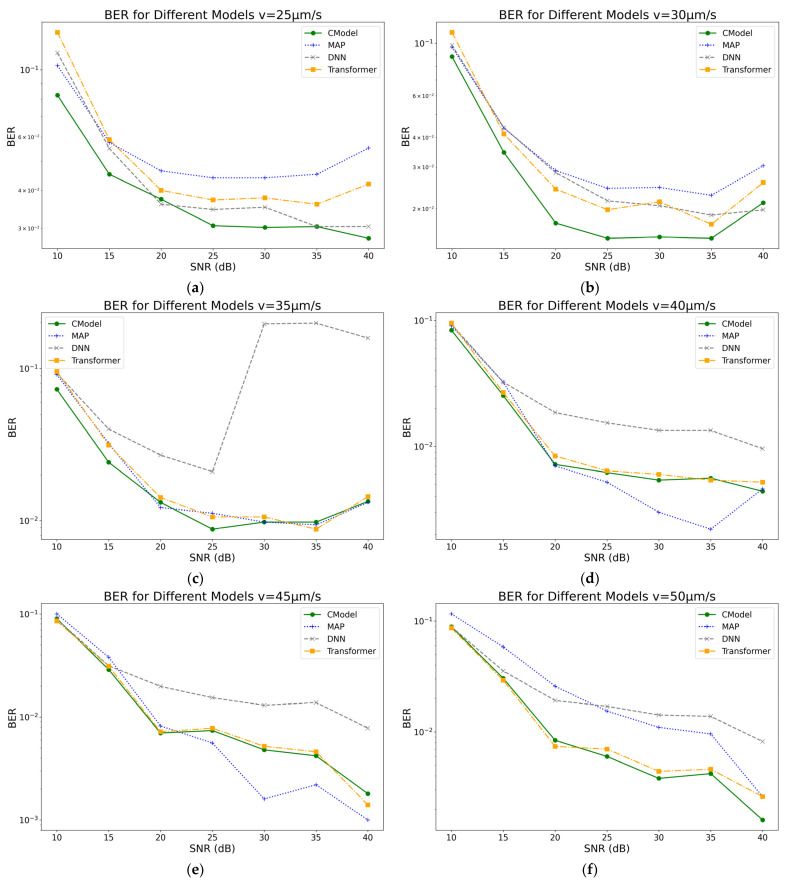
BER of different models at various SNR values for different drift velocities: (**a**) v=25 μm/s; (**b**) v=30 μm/s; (**c**) v=35 μm/s; (**d**) v=40 μm/s; (**e**) v=45 μm/s; (**f**) v=50 μm/s. The results show that CModel exhibits the most stable and lowest BER across all SNR levels. As SNR increases, the BER of CModel decreases significantly and remains lower than all other models, demonstrating clear superiority.

Noise affects the number of received molecules $n_{i}$, leading to variations in the observed values. The CModel robustly handles these variations through positional embedding and residual connections. Under different SNR conditions, the BER of the CModel, MAP, DNN, and Transformer models is calculated, and the performance of each model in communication systems subjected to various noise environments is recorded. The BER performance of these models across different SNR ranges is compared at v=25 μm/s, 30 μm/s,35 μm/s, 40 μm/s, 45 μm/s, 50 μm/s. The experimental results are presented in Figure 5.

As shown in Figure 5, overall, the BER further decreases with increasing drift velocity. The MAP model, which is based on complete channel statistics, achieves the lowest BER under low SNR conditions, approximately 0.12. As SNR increases, its BER rapidly declines, reaching a minimum of about 0.03 at 20 dB. However, when the SNR further increases to the range of 30–40 dB, the BER of the MAP model exhibits fluctuations and a slight increase, reflecting its limited adaptability in complex and dynamic noise environments. Since the noise environment in practical MC systems is often unknown and complex, the MAP model cannot accurately compute the posterior probability under such conditions, resulting in performance limitations and making its BER inferior to deep learning-based models.

In contrast, the CModel demonstrates more stable and superior performance. Although its BER at 10 dB is slightly higher than that of MAP, at approximately 0.11, it drops rapidly to 0.07 at 15 dB and further decreases to around 0.03 at 20 dB. The BER continues to fall with increasing SNR, reaching approximately 0.007 at 40 dB, maintaining the lowest level among all models. This excellent performance is mainly attributed to its outstanding feature extraction capability and effective noise suppression mechanisms, which provide high robustness and accuracy in medium and high SNR ranges.

Meanwhile, the DNN model exhibits relatively high BER across the entire SNR range, with an initial value of about 0.15. Although its BER gradually decreases with increasing SNR, it still remains as high as 0.07 at 30 dB and does not fall below 0.03 at 40 dB, indicating its limited noise suppression ability under complex channel conditions. The BER of the Transformer model fluctuates considerably in the low SNR range, between 0.1 and 0.12. However, as SNR increases, the BER gradually decreases, reaching approximately 0.05 at 30 dB and approaching 0.015 at 40 dB. In terms of overall performance, the Transformer outperforms the DNN but still lags behind CModel and exhibits poor stability under low SNR conditions.

In Figure 5, the BER tends to converge as SNR increases, primarily due to the shift in dominant system errors in the high SNR regime from readout thermal noise (whose Gaussian white noise impact diminishes rapidly) to channel-intrinsic uncertainties, such as Poisson counting noise and ISI caused by diffusion tails, leading to an irreducible error floor that cannot be further reduced.

The influence of drift velocity on BER is also significant. At higher drift velocities, for example, the average BER of all models decreases by about 10% compared to lower velocities, indicating that accelerating molecular transmission rates reduces signal diffusion and blurring, which benefits fast and accurate information detection.

In summary, the experiments clearly demonstrate that the proposed CModel maintains consistently low and robust BER performance across various SNR and drift velocity combinations.

## 5. Discussion

This study systematically demonstrates that the proposed CModel achieves excellent and stable detection performance across various drift velocities and SNR conditions, highlighting its strong capability for molecular signal feature extraction. Compared to conventional detection techniques, CModel exhibits superior applicability in practical and complex MC scenarios. Notably, CModel achieves faster convergence during training and maintains higher detection accuracy under different drift and SNR conditions, thereby outperforming mainstream DNN models. Experimental results show that baseline models such as DNN and Transformer experience significant performance degradation as drift velocity increases or noise levels rise. In contrast, CModel is able to maintain outstanding stability and achieve extremely low BERs even under medium-to-high drift and high-noise environments. This remarkable advantage can be attributed to CModel’s ability to effectively extract both local sequential features and global contextual dependencies—two aspects that are difficult to balance in traditional models.

Recently, some studies have explored integrating attention mechanisms or convolutional structures into sequence detection for MC. However, approaches relying on standard self-attention mechanisms (such as those used in Transformers) often exhibit high computational complexity for long sequences, while purely convolutional methods are limited in modeling long-range dependencies. CModel addresses these issues by leveraging the probSparse attention mechanism to significantly reduce computational burden and by incorporating cross-attention and convolutional modules to enable multi-scale, context-aware feature extraction. Compared to models such as Transformer and MAP-based detectors, CModel achieves a better balance among detection accuracy, computational efficiency, and adaptability to diverse channel conditions. The specific advantages of CModel in MC systems are manifested in multiple aspects: First, it provides efficient modeling for long-tail ISI, capable of handling long-tail crosstalk across multiple symbol intervals caused by diffusion–drift, through Informer’s sparse attention and segmental modeling to selectively model long dependencies at near-linear complexity, which is more computationally efficient than traditional Transformers and more flexible than fixed-order equalization or convolutional methods; additionally, it exhibits robustness to statistical mismatch and non-stationary noise, as practical MC often involves non-pure Poisson processes, time-varying background rates, and external interferences, while CModel, through noise consistency, gating, and multi-condition joint training, weakens the dependence on precise distribution assumptions, maintaining stable BER under unknown or mismatched noise; simultaneously, the attention mechanism can adaptively focus on key time slices (such as delay windows with high hit probabilities) and suppress irrelevant intervals, offering better adaptability to scenarios like varying drift velocities and diffusion coefficients.

Despite these advantages, the architectural complexity of CModel leads to higher training times and greater computational requirements, which may pose challenges for deployment in resource-constrained or real-time applications. In addition, the fixed kernel size in the convolutional module is beneficial for capturing local dependencies, but it limits the model’s ability to extract multi-scale features, which are inherent in practical MC signals. The performance of CModel is also sensitive to hyperparameter configurations, including dropout rates and attention mechanism parameters, thus requiring extensive tuning and empirical validation.

In summary, the proposed CModel represents a significant advance over existing molecular signal detection solutions. This approach overcomes key limitations of traditional methods and achieves superior detection accuracy and robustness under complex and dynamic channel conditions.

## 6. Conclusions

In this study, the proposed CModel incorporates probSparse Attention, Cross Attention, and convolutional layers, significantly enhancing both sequence modeling and signal detection capabilities. The experimental results demonstrate that CModel exhibits rapid convergence during training, consistently achieving recognition accuracy above 99% across a variety of environments and drift velocities. Notably, under medium and high SNR conditions, the BER can be reduced to 0.007, which is markedly lower than those achieved by conventional models such as DNN, Transformer, and MAP, thereby highlighting its robust adaptability to diverse conditions.

This research bears substantial practical significance, as CModel maintains stable detection accuracy in complex and dynamic MC environments. Its exceptional feature extraction and noise suppression capabilities contribute to promising prospects for real-world applications. In this work, we adopt a diffusion model based on Fick’s diffusion law as the baseline for the MC channel, benefiting from its physical interpretability and analytical tractability in end-to-end detection. Meanwhile, future work will extend to more realistic scenarios, such as reaction–diffusion models and confined geometries, and focus on incorporating more efficient attention mechanisms, optimizing multi-scale convolutional architectures, developing realistic testing platforms, and leveraging advanced regularization, pre-training, and hyperparameter refinement techniques to further enhance the generalization, robustness, and practical deployment of CModel in complex real-world scenarios. In summary, CModel achieves efficient and resilient sequence detection in MC signal detection tasks, providing a viable solution for advancing deep learning applications in related fields.

## Figures and Tables

**Table 1:** Overview of related work.

Author/Reference	Method	Application Scenario	Results
Yue et al. [12]	Derived a mathematical model for channel impulse response based on microcirculation networks (MCNs) and blood flow characteristics, constructed an end-to-end communication model	Channel modeling and error probability analysis in MC systems	Analyzed error probability, providing accurate system descriptions
Farsad et al. [13]	Sliding Bidirectional Recurrent Neural Network (SBRNN) detection technique, estimates received signal flow in real time through training without relying on underlying channel model	Real-time signal detection in MC systems, adapting to rapidly changing channel environments	BER outperforms detectors with imperfect channel state information (CSI) and other neural network detectors, performs well under various channel conditions
Gomez et al. [14]	Interpretable deep learning symbol detection method	MC signal detection based on real test platform data	Provided interpretable symbol detection performance
Bartunik et al. [15]	Convolutional Neural Network (CNN) signal demodulation method	Scenarios with varying channel parameters in MC, requiring minimal channel knowledge	Achieved 1% classification accuracy at high symbol rates, significantly outperforming linear discriminant analysis
Bai et al. [16]	Improved time series convolutional network	Signal detection in specific mobile communication systems	Improved signal detection performance
Lee et al. [17]	Artificial Neural Network (ANN) to predict MC channel parameters, trained using nonlinear least squares curve fitting based on simulation data	Prediction of MC channel parameters based on simulation data	Effectively predicted channel parameters
Baydas et al. [18]	CNN and Recurrent Neural Networks (RNN)	BER performance evaluation in MC systems	Evaluated BER performance, demonstrating the potential of deep learning in MC
Lu et al. [19] and Cheng et al. [20]	Transformer detection model	Signal detection in MC	Performance significantly surpasses that of DNN detectors
Khalopour et al. [21]	Analyzed performance of fusion centers and gateways	Perception, communication, and localization in MC systems under ideal (noise-free) and non-ideal (noisy) channel conditions	Evaluated system performance under different channel conditions

**Table 2:** The parameters of MC system [19].

Parameter	Symbol	Value
Diffusion coefficient	D	$79.4\;{\mu m}^{2}/s$
Velocity	v	$\left\{25,30,35,40,45,50\right\}\;\mu m/s$
Distance between TN and RN	d	10 μm
Radius of RN	*R*	1.5 μm
Released molecules per release time	M	4000
Precision control parameter	ϵ	$10^{-2}$

**Table 3:** The parameters of CModel.

Parameter	Value
Encoder Layers	3
Decoder Layers	3
Attention Heads	8
Hidden Layer Dimension	128
Dropout Parameter	0.1
Training Epochs	500
Batch Size	60

## Data Availability

The data presented in this study are available on request from the corresponding author. The data are not publicly available due to privacy.
